# The Impact of Ursodeoxycholic Acid on Fetal Cardiac Function in Women with Gestational Diabetes Mellitus: A Randomized Controlled Study (GUARDS Trial)

**DOI:** 10.3390/jcm14207366

**Published:** 2025-10-17

**Authors:** Ana Maria Company Calabuig, Jose Eliseo Blanco Carnero, Christos Chatzakis, Catherine Williamson, Kypros H. Nicolaides, Marietta Charakida, Catalina De Paco Matallana

**Affiliations:** 1 Department of Obstetrics and Gynecology, Hospital Clínico Universitario Virgen de la Arrixaca, El Palmar, 30120 Murcia, Spain; anamariacompanycalabuig@gmail.com (A.M.C.C.); jeblancoc@gmail.com (J.E.B.C.); 2 Faculty of Medicine, Universidad de Murcia, 30107 Murcia, Spain; 3 Biomedical Research Institute of Murcia Pascual Parrilla—IMIB, 30120 Murcia, Spain; 4 Harris Birthright Research Centre for Fetal Medicine, King’s College London, London SE5 8BB, UK; cchatzakis@gmail.com (C.C.); kypros@fetalmedicine.org (K.H.N.); charakidadoc@googlemail.com (M.C.); 5 Institute of Reproductive and Developmental Biology, Department of Metabolism, Digestion & Reproduction, Imperial College London, London W12 0NN, UK; catherine.williamson@imperial.ac.uk; 6 School of Biomedical Engineering and Imaging Sciences, King’s College London, London SE1 7EH, UK

**Keywords:** gestational diabetes mellitus (GDM), bile acids, ursodeoxycholic acid, trial, fetal cardiac function, pregnancy outcome

## Abstract

**Background:** Gestational diabetes mellitus (GDM) is associated with subclinical alterations in fetal cardiac morphology and function. Ursodeoxycholic acid (UDCA), widely used in pregnancy for intrahepatic cholestasis, has demonstrated cardioprotective properties in experimental fetal models, preventing conduction abnormalities and improving myocardial function. Whether UDCA modifies fetal or neonatal cardiac adaptation in GDM pregnancies has not been previously investigated. The objective was to evaluate the effect of ursodeoxycholic acid (UDCA) on fetal and neonatal cardiac function in pregnancies complicated by gestational diabetes mellitus (GDM). **Methods**: In this randomized, placebo-controlled study, 113 women with GDM were enrolled, of whom 56 received UDCA and 57 the placebo. After measurement of maternal blood UDCA concentrations, 43 participants in the treatment group had levels ≥0.5 µmol/L and were included in the per-protocol analysis. Echocardiographic and Doppler-derived cardiac indices were assessed at baseline, 36 weeks’ gestation, and postpartum. Comparisons were performed using univariable tests and mixed-effects multivariable models accounting for time and treatment. **Results:** In the treatment group, compared to the placebo group, there were no significant differences in cardiac indices at 36 weeks’ gestation or postpartum when assessed individually. However, in the mixed-effects longitudinal analysis, a significant treatment-by-time interaction was observed. Specifically, in the postpartum period, mitral A-wave velocity (MV-A) was higher in the treatment group compared to that under the placebo (9.58, 95% CI 2.29–16.87; *p* = 0.010), reflecting a more pronounced increase in the atrial contribution to left ventricular filling over time. Similarly, aortic peak velocity (Ao_Vmáx) was significantly higher in the treatment group compared to that under the placebo in the postpartum period (7.97, 95% CI 0.19–15.75; *p* = 0.045), indicating a greater augmentation in left ventricular outflow dynamics. **Conclusions**: In pregnancies complicated by GDM, UDCA did not lead to significant cross-sectional differences in fetal or neonatal cardiac indices at 36 weeks or postpartum. However, longitudinal modeling indicated that UDCA was associated with a greater increase in the atrial contribution to ventricular filling (MV-A) and aortic peak velocity (Ao_Vmáx) in the postpartum period compared to that under the placebo. These findings suggest that while UDCA does not broadly alter cardiac function, it may modulate specific aspects of diastolic filling and systolic outflow dynamics during late gestation and early neonatal adaptation.

## 1. Introduction

Gestational diabetes mellitus (GDM) is a common metabolic disorder during pregnancy characterized by glucose intolerance, which can lead to adverse outcomes for both the mother and child, including macrosomia, shoulder dystocia, neonatal hypoglycemia, and long-term metabolic diseases in the offspring [1,2]. Globally, the prevalence of GDM ranges between 5% and 20%, depending on various factors, and it is increasing in parallel with maternal age, obesity, and a sedentary lifestyle [1]. Women with GDM are seven times more likely to develop type 2 diabetes later in life, and their offspring are at an increased risk of obesity, metabolic syndrome, and cardiovascular disease [1,2]. Thus, GDM not only represents a serious problem during pregnancy but is also a major risk for developing a cardiometabolic disease beyond pregnancy.

Beyond this, there is increasing attention on the impact of GDM on the fetal cardiovascular system. Our group and others have observed that the fetuses of mothers with GDM show structural and functional cardiac alterations, including interventricular septal hypertrophy, increased myocardial mass, impaired diastolic relaxation, and an abnormal global myocardial performance index. Aguilera et al. found that women with GDM and their fetuses both had subclinical cardiac dysfunction in the third trimester, with fetuses demonstrating more globular-shaped hearts with increased right and left ventricular sphericity indices and a reduced global systolic performance [3]. Yovera et al. showed that GDM was associated with significant changes in fetal cardiac morphology and function between the second and third trimesters, including impaired right ventricular function and an abnormal global sphericity index [4]. More recently, Huluta et al. reported that even at midgestation and prior to the diagnosis of GDM, fetuses already showed early mild increases in septal thickness and left atrial area, although systolic and diastolic function remained preserved [5]. These studies support the concept of early diabetic cardiomyopathy in utero, which may contribute to long-term cardiovascular risk in exposed offspring.

Oxidative stress, increased myocardial workload, altered glucose and lipid metabolism, and prolonged fetal hyperinsulinemia are considered the pathophysiological reasons for these cardiac changes. Excessive glucose transfer across the placenta leads to increased fetal insulin secretion, which is a growth factor for the heart that promotes septal and ventricular hypertrophy [3,4,5]. At the same time, exposure to inflammatory and oxidative mediators may affect cardiovascular risk, which persists into infancy and adulthood and is likely influenced by these intrauterine programming mechanisms [1,2].

Several mechanisms might explain these cardiac changes, including oxidative stress, increased myocardial workload, altered glucose and lipid metabolism, and prolonged fetal hyperinsulinemia. In addition, exposure to inflammatory and oxidative mediators may impair myocardial structure and function further, contributing to long-term cardiovascular risk. This risk, which persists into infancy and adulthood, is likely influenced by intrauterine programming mechanisms [1,2]. Experimental and human cohort studies demonstrate that perturbations in the intrauterine metabolic environment, such as those associated with maternal diabetes and cholestasis, predispose offspring to long-term metabolic and cardiovascular diseases via epigenetic modifications and altered lipid handling [6]. These findings suggest that early exposure to hyperglycemia and metabolic imbalance can induce persistent cardiovascular vulnerability.

Ursodeoxycholic acid (UDCA) is a hydrophilic bile acid that is used to treat intrahepatic cholestasis of pregnancy (ICP), improving maternal symptoms and biochemical parameters and showing a good safety profile. Beyond hepatoprotection, UDCA has antioxidant, anti-apoptotic, anti-inflammatory, and mitochondrial stabilizing properties [7]. In preclinical studies, UDCA has been shown to have the ability to reduce myocardial fibrosis through TGR5 receptor signaling [7], preserve mitochondrial integrity, and limit apoptosis in cardiomyocytes. These actions are enhanced by antiarrhythmic effects: UDCA restores T-type calcium currents, inhibits ventricular conduction slowing, and reduces arrhythmogenesis induced by cholestatic bile acids [8,9]. Importantly, UDCA also affects cardiac fibroblasts by stopping the remodeling of the extracellular matrix in a way that makes it less flexible, thereby contributing to improved myocardial compliance [10].

Interestingly, UDCA has also been linked to cardioprotective effects. Experimental studies indicate that whilst taurocholic acid disrupts ventricular conduction and induces fetal arrhythmias in ICP, UDCA counteracts these effects by normalizing calcium currents and regulating myofibroblast activity [7,8]. Other studies have shown that fetuses from mothers with intrahepatic cholestasis have prolonged atrioventricular conduction intervals, which decrease following maternal UDCA therapy, particularly in severe cases [9,11].

Recent studies have expanded the potential cardiovascular applications of UDCA. In pregnant women with ICP, treatment with UDCA showed a reduction in fetal dyslipidemia, an improvement in glucose tolerance in adult offspring, and even the reversal of epigenetic alterations associated with metabolic disease susceptibility [10]. Whilst taurocholic acid disrupts ventricular conduction and induces fetal arrhythmias in intrahepatic cholestasis of pregnancy (ICP), UDCA mitigates these effects by normalizing calcium currents and regulating myofibroblast activity [7,8]. Additionally, fetuses from mothers with ICP had prolonged atrioventricular conduction intervals, which decreased following maternal UDCA therapy, particularly in severe cases [9,11]. Reilly-O’Donnell et al. observed that UDCA reduces interstitial fibrosis via TGR5 signaling and improves conduction velocity in vitro and ex vivo and therefore decreases arrhythmic vulnerability [6,12]. In a chronic post-myocardial infarction model, UDCA further attenuated ventricular remodeling, preserved systolic function, and reduced fibrosis in the infarct border zone, with computational simulations confirming a reduction in arrhythmia inducibility [12].

Despite the impact of GDM on fetal cardiac function, no pharmacological interventions have yet been shown to decrease these effects. Considering the metabolic and circulatory effects of UDCA, this may be considered a promising candidate for further research.

The aim of this study was therefore to evaluate the effect of UDCA compared with that of a placebo on fetal cardiac function in pregnancies complicated by GDM.

## 2. Materials and Methods

### 2.1. Study Design and Participants

This prospective study was carried out within the GUARDS trial (a randomized, controlled trial of the impact of UDCA on glycemia in GDM) [13]. This randomized, placebo-controlled, double-blind trial (IMIB-GU-2019-02, date of registration: 17 June 2020; date on which the first participant was enrolled: 3 March 2021) was performed at Clinic University Hospital, “Virgen de la Arrixaca”, in Murcia, Spain.

#### Sample Size Estimation

The initial calculations indicated that 204 participants would be sufficient to provide 90% power to detect a significant reduction in maternal fasting glucose at 36 weeks, while accounting for a 40% withdrawal rate (the primary outcome of the GUARDS trial). This required 61 women per arm, which increased to 204 in total after allowing for drop-outs.

Due to COVID-related delays and limited availability of the placebo, updated calculations were performed. By the time the investigational product expired, 113 participants had been recruited, with 5 withdrawing. Revised estimates showed that 50 women per group would provide 82% power, and 55 women per group would provide 85% power, both at a significance level of *p* < 0.05.

**Figure 1:**
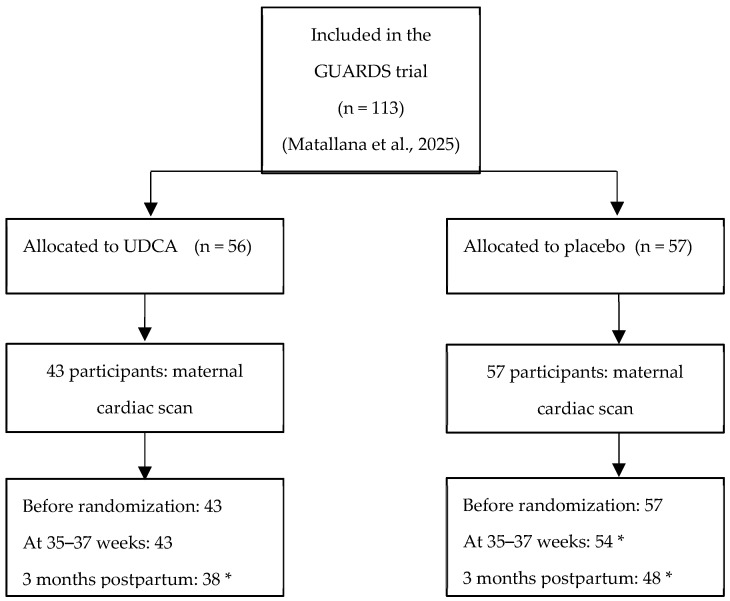
Flow diagram of participant inclusion. Adapted from [13,14]. * Lost to follow-up.

Recruitment and eligibility data were reported in a CONSORT flow diagram (Figure 1).

Women with an abnormal oral glucose tolerance test (OGTT) screen were offered the chance to participate between 24 + 0 and 30 + 6 weeks’ gestation. In Spain, a diagnosis of GDM involves a two-step screening process: Step 1: The O’Sullivan test—conducted between 24 and 28 weeks of gestation. A 1 h plasma glucose level ≥ 140 mg/dL (7.8 mmol/L) after a 50 g glucose load is considered positive. Step 2: A 100 g 3 h OGTT—performed after an overnight fast. The diagnosis of GDM is confirmed if at least two of the following thresholds are met or exceeded: fasting: ≥95 mg/dL (5.3 mmol/L); 1 h: ≥180 mg/dL (10.0 mmol/L); 2 h: ≥155 mg/dL (8.6 mmol/L); 3 h: ≥140 mg/dL (7.8 mmol/L) [1,2].

Inclusion criteria were GDM diagnosed at 24–28 weeks’ gestation; planned antenatal care at the same center; a singleton pregnancy; and the ability to give informed and written consent. The exclusion criteria included an age < 18 years; a previous diagnosis of diabetes outside of pregnancy; significant pre-pregnancy comorbidities that increase risk in pregnancy; and significant comorbidity in the current pregnancy.

After providing written informed consent, eligible women were randomly assigned into the group receiving treatment with UDCA 500 mg orally twice daily or the group receiving the placebo until the time of delivery. In the present study, women that were allocated either into the UDCA group or the placebo group agreed to have a cardiac scan on the day of randomization and before starting medication, as well as at 35–37 weeks and at 3 months postpartum.

The study was conducted in accordance with the Declaration of Helsinki, and the protocol was reviewed and approved by the Ethics Committee of Murcia (code 2018-11-5-HCUVA).

### 2.2. Maternal and Fetal Characteristics

We recorded information on maternal age, ethnic origin (White, Black, Asian, or mixed), method of conception (spontaneous or assisted through in vitro fertilization or ovulation induction drugs), cigarette smoking, and parity (parous or nulliparous if there was no previous pregnancy with delivery at ≥24 weeks of gestation). At every clinic visit, weight and height were measured, and body mass index was calculated. At every visit, a prenatal ultrasonographic examination was performed to estimate fetal weight from measurements of fetal head circumference, abdominal circumference, and femur length (EPIQ Elite Philips, Bothell, WA, USA) [15], and the values were converted into Z-scores based on the Fetal Medicine Foundation’s fetal weight chart [16].

### 2.3. Fetal Cardiac Functional Analysis

Fetal cardiac functional measurements were performed at an ‘apex oblique’ projection with an angulation of at least 30° (EPIQ Elite Philips C5-1 or C9 transducer). Cardiac function was assessed using conventional Doppler. Fetal heart rate was calculated using spectral Doppler imaging of the aortic flow [17]. Left ventricular outflow tract diameter (LVOTd) and peak aortic velocity (Ao Vmax) were obtained, and ejection time (ET) was measured from Doppler waveforms of the aortic outflow. The left myocardial performance index (MPI) was calculated using pulsed-wave Doppler in a five-chamber view, with the sample volume including both the aortic and mitral flows; valvular clicks in the Doppler waveforms were used as landmarks to define each time interval [18]. Systolic function was evaluated by measuring tricuspid annular plane systolic excursion (TAPSE) using the M-mode and the isovolumetric contraction time (IVCT) from Doppler waveforms. Diastolic function was assessed by measuring the peak early (E) and late (A) ventricular filling velocities across the mitral valve, as well as the isovolumetric relaxation time (IVRT), using Doppler flow patterns. The E/A ratio was subsequently calculated [17].

### 2.4. Statistical Analysis

Continuous variables were presented as the mean  ±  SD if their distribution was normal or as the median (interquartile range) if their distribution was non-normal. Normality of the distribution was tested using the Kolmogorov–Smirnov test. Categorical variables were presented as n (%). Individual comparison between the placebo and treatment groups at the late third trimester and postpartum was performed using the *t*-test or the Mann–Whitney U test. *p*-values are presented for direct two-way comparisons between groups. *p*  <  0.05 was considered statistically significant.

Linear mixed models were fitted to assess the effect of time and treatment on maternal cardiac indices after accounting for maternal characteristics. The statistical software package R (version 4.5.1; R Foundation for Statistical Computing, Vienna, Austria) was used for data analysis [19] (see Supplementary Tables S1–S7).

## 3. Results

### 3.1. Participant Characteristics

In the current study, 113 patients were included, of whom 56 received UDCA and 57 received a placebo. After assessing the levels of UDCA in the maternal blood, 43 of the 56 had blood concentrations ≥ 0.5 µmol/L (consistent with having taken and absorbed the drug) and were included in the analysis.

There were no significant differences between groups with regard to participant characteristics and cardiac functional indices prior to randomization, with the exception of Ao_Vmáx. Individual comparisons between the placebo and treatment group are shown in Table 1 and Table 2.

### 3.2. Comparison of Cardiac Indices During the Study

Individual comparisons between the treatment and placebo groups at 36 weeks’ gestation and postpartum did not show significant differences in the cardiac indices (Table 2). However, the multivariable analysis using mixed models to account for differences in time and treatment indicated that MV-A and Ao_Vmáx increased over time in both the treatment group and the control group. However, this increase was more pronounced in the treatment group.

Specifically, there was a significant interaction for MV-A between treatment and time in the postpartum period (9.58; *p* = 0.010). In addition, there was a significant interaction for Ao_Vmáx between treatment and time in the postpartum period (7.97; *p* = 0.045) (Table 3 and Table 4).

## 4. Discussion

### 4.1. Principal Findings

The findings of this randomized controlled trial suggest that ursodeoxycholic acid (UDCA) may exert a modest but specific effect on fetal and neonatal cardiac functional indices in pregnancies complicated by GDM. While the cross-sectional comparisons at 36 weeks and postpartum did not reveal significant differences between groups, the longitudinal mixed-model analysis demonstrated a significant treatment-by-time interaction postpartum: the UDCA group was associated with greater increases in mitral A-wave velocity (MV-A) and aortic peak velocity (Ao_Vmáx) in the postpartum period compared to those under the placebo. These findings suggest that UDCA may have a selective effect on the atrial contribution to ventricular filling and systolic outflow dynamics, rather than broad changes in global cardiac function.

### 4.2. Results in the Context of What We Know

#### Fetal Cardiac Changes in GDM

Multiple studies have shown that GDM is associated with subclinical remodeling—septal thickening, altered geometry (reduced global sphericity), and subtle functional changes (impaired diastolic relaxation; RV functional reduction), even when glycemia is apparently controlled. In the third trimester, Aguilera et al. demonstrated paired maternal–fetal alterations, including more globular fetal hearts and a reduced global systolic performance, in the second and third trimesters [3]. Yovera et al. reported reduced RV function and altered sphericity [4], and in the second trimester, Huluta et al. identified early morphological changes preceding GDM diagnosis with largely preserved function [5].

Our finding that MV-A increased more in the UDCA-treated pregnancies suggests that UDCA affects late diastolic filling, an essential component of ventricular relaxation. This is especially important in GDM, where subtle impairments in diastolic function are common, and this supports the hypothesis that UDCA may modulate the atrial–ventricular interplay during fetal cardiac adaptation. Significantly, our findings show that UDCA does not affect MPI or TAPSE, supporting the hypothesis that UDCA’s effects may be selective.

### 4.3. UDCA and the Fetal Heart

UDCA is widely used for intrahepatic cholestasis during pregnancy and has shown cardioprotective properties in both experimental and clinical contexts. Some studies have demonstrated that UDCA inhibits bile-acid-induced conduction slowing, restores T-type calcium currents, and reduces arrhythmias in fetal heart models [8,11]. Clinical observations in ICP indicate that UDCA reduces the fetal atrioventricular conduction intervals, especially in severe cases [9]. Vasavan et al. also showed that increased maternal bile acid levels in ICP were directly associated with fetal cardiac dysfunction, highlighting the distinct cardiotoxicity of the primary bile acids that are principally raised in untreated ICP, i.e., glyco- and tauro-conjugated cholic acid [20].

In line with this, our findings suggest that UDCA may also influence fetal diastolic filling (via MV-A) and systolic outflow (via Ao_Vmáx) in GDM pregnancies, rather than changes in conduction. The increase in Ao_Vmáx may improve forward stroke dynamics, while the rise in MV-A suggests greater atrial–ventricular coupling during adaptation. Recent findings by Chivers et al. further support that GDM pregnancies are associated with an altered fetal heart rate and autonomic regulation, strengthening the concept that the fetal heart is functionally susceptible in this metabolic context [21].

Differences in study populations (GDM vs. ICP), exposure patterns (hyperglycemia vs. elevated bile acids), and the specific endpoints evaluated (functional indices vs. conduction markers) likely explain why we did not observe MPI changes in our GDM cohort. This aligns with aggregate findings from Depla et al., who reported no MPI difference in GDM fetuses despite consistent signs of diastolic dysfunction [22], and from Skovsgaard et al., which noted inconsistent MPI outcomes in neonatal cardiac studies of diabetic pregnancies [23]. In contrast, cardiac dysfunction in ICP is more consistently manifested through conduction abnormalities and global indices such as the MPI. This was observed by Vasavan et al., who proved that elevated maternal bile acid concentrations were directly associated with fetal cardiac dysfunction, particularly atrioventricular conduction disturbances [20]. Zhan et al. also obtained the same results in a meta-analysis, reinforcing the evidence of a cardiotoxic effect of bile acids on the fetal heart [24].

### 4.4. Clinical and Research Implications

The findings of this study suggest that UDCA may have a modest, time-dependent influence on fetal and neonatal cardiac function in pregnancies complicated by GDM, selectively affecting indices of atrial filling (MV-A) and systolic outflow (Ao_Vmáx). Although these effects were statistically significant, they were limited to specific parameters and were not accompanied by broader improvements in systolic or global function. Therefore, their clinical relevance remains uncertain.

From a clinical standpoint, the lack of negative impacts on cardiac function offers reassurance regarding the safety of UDCA administration in this population. Given that offspring of GDM pregnancies are more likely to have cardiac and metabolic disorders later in life, even small improvements in fetal cardiac function could be important, but this hypothesis requires validation. In addition, while studies in ICP have consistently shown that UDCA mitigates conduction abnormalities and reduces the global dysfunction caused by elevated bile acids [20,24], our data suggest that in GDM, UDCA may act through different pathways, modulating functional Doppler indices rather than conduction. At present, our results should be interpreted as preliminary and not as evidence supporting clinical benefit. Larger and adequately powered longitudinal studies are needed to incorporate not only echocardiographic measures but also maternal metabolic and inflammatory markers to clarify potential mechanisms. Future work should explore whether UDCA’s effects in GDM reflect direct myocardial actions or indirect modulation via improved metabolic or vascular function and whether such selective changes can contribute to longer-term cardiovascular outcomes in affected offspring.

In this context, the integration of recent preclinical and translational evidence is important. The improvements in fetal lipid metabolism and protection against adverse metabolic programming observed in women with ICP in treatment with UDCA [6], together with its antifibrotic and antiarrhythmic effects demonstrated in experimental cardiac models [7,12], highlight the need for further research.

### 4.5. Strengths and Limitations

A major strength of this study is its randomized, placebo-controlled design, which allows for the assessment of both systolic and diastolic performance. Compliance was carefully monitored through maternal blood UDCA concentrations, ensuring an accurate per-protocol analysis. Nevertheless, several limitations should be acknowledged. First, the sample size was relatively small, limiting the statistical power. Second, significant differences were confined to two indices (MV-A and Ao_Vmáx), while the majority of the functional measures remained unchanged; this selective pattern raises uncertainty regarding the consistency and physiological significance of the findings. Third, echocardiographic indices, despite their sensitivity to functional changes, may not directly correlate with clinically significant outcomes, and this study was not powered to assess perinatal or long-term cardiovascular endpoints. Finally, the mechanisms underlying the observed associations remain unclear, and it is uncertain whether they reflect direct myocardial actions of UDCA or indirect effects mediated by maternal metabolic or vascular changes.

## 5. Conclusions

UDCA administration in mid-pregnancy to women with GDM was associated with selective increases in MV-A and Ao_Vmáx during late gestation and postpartum, while the other functional indices remained unchanged. These results should be interpreted cautiously: the observed changes were modest and confined to specific parameters, and their clinical significance remains uncertain. Larger, longer-term studies are needed to confirm these findings, clarify underlying mechanisms, and determine whether such selective effects translate into meaningful clinical benefit.

## Figures and Tables

**Table 1:** Baseline participant characteristics by randomized treatment group.

	UDCA (43)	Placebo (57)	*p* Value
Maternal age (years)	34.5 (4.31)	32.6 (6.11)	0.071
BMI (kg/m^2^)	29.6 (3.98)	29.4 (4.50)	0.764
Weight (kg)	78.2 (12.9)	77.9 (13.5)	0.922
Gestational age (days)	27.9 (1.71)	28.2 (1.75)	0.303
Ethnicity			0.887
-White	42 (96.4%)	57 (100%)	
-Asian	1 (2.3%)	0 (0%)	
Nulliparity	22 (51.1%)	22 (38.5%)	0.487
Fasting glucose at OGTT (mg/dL)	87.1 (14.3)	87.8 12.4)	0.802
1 h glucose at OGTT (mg/dL)	192.0 (27.0)	194.0 (21.4)	0.747
2 h glucose at OGTT (mg/dL)	182.0 (23.0)	185.0 (17.1)	0.671
HbA1c (%)	5.15 (0.34)	5.22 (0.50)	0.439
Biochemical profile			
Total cholesterol (mg/dL)	236.0 (47.9)	229.0 (37.4)	0.503
HDL-cholesterol (mg/dL)	70.2 (12.1)	72.9 (17.1)	0.375
LDL-cholesterol (mg/dL)	122.0 (41.1)	116.0 (35.4)	0.518
Triglycerides (mg/dL)	216.0 (68.9)	200.0 (49.0)	0.221
CRP (mg/dL)	0.61 (0.51)	0.70 (0.74)	0.514
Ratio (s-Flt1:PLGF)	2.7 (2.1)	2.5 (2.3)	0.847
ALT/GOT (IU/L)	12.6 (6.04)	13.1 (6.38)	0.325
AST/GPT (IU/L)	14.7 (3.35)	15.6 5.29)	0.711

Results are presented as the mean (standard deviation) where continuous variables and the number affected (n)/total number in group (N) where qualitative variables. Comparisons between the groups were carried out using the *t*-test for continuous variable and the chi-square test or Fisher’s exact test for qualitative variables. BMI: body mass index; OGTT: oral glucose tolerance test; HDL: high-density lipoprotein; LDL: low-density lipoprotein; CRP: C-reactive protein; s-Flt: soluble FMS-like tyrosine kinase 1; PLGF: placental growth factor; ALT: alanine aminotransferase; GOT: glutamic oxaloacetic transaminase; AST: aspartate aminotransferase; GPT: glutamic pyruvic transaminase.

**Table 2:** Echocardiographic parameters at baseline, third trimester, and postpartum in control and intervention groups.

Timepoint	Variable	Mean Control	SD Control	Mean Intervention	SD Intervention	*p* Value
**Baseline**						
	Ao_Vmáx_(Doppler)	77.3	13.5	71.6	12.8	0.0365
	ET_(msec)	165.0	13.9	165.0	27.7	0.95
	HR	144.0	12.4	142.0	10.8	0.409
	IVCT_(msec)	40.4	8.13	43.9	9.45	0.059
	IVRT_(msec)	51.5	9.59	55.3	13.3	0.113
	LVOT_d	4.65	0.682	4.62	0.611	0.815
	MPI	40.6	7.95	43.7	9.52	0.0846
	MV_A	50.5	9.61	48.3	9.7	0.252
	MV_E	35.7	8.35	34.6	6.34	0.449
	TAPSE_(mm)	5.86	1.11	5.78	1.12	0.723
	TV_A	52.2	9.88	51.0	9.77	0.589
	TV_E	41.0	10.5	41.4	10.9	0.856
**Third** **Trimester**						
	Ao_Vmáx_(Doppler)	83.1	16.8	84.7	15.9	0.639
	ET_(msec)	168.0	15.0	166.0	15.8	0.616
	HR	140.0	14.7	141.0	11.1	0.677
	IVCT_(msec)	41.5	10.4	41.4	7.55	0.971
	IVRT_(msec)	57.9	10.5	59.6	11.7	0.471
	LVOT_d	6.13	0.801	6.04	0.777	0.586
	MPI	41.3	9.73	41.3	7.16	0.985
	MV_A	50.3	9.8	49.4	8.77	0.631
	MV_E	39.9	8.24	40.4	8.79	0.78
	TAPSE_(mm)	7.27	1.05	8.5	5.89	0.184
	TV_A	60.3	8.87	55.7	11.9	0.0497
	TV_E	47.7	10.3	49.7	13.0	0.405
**Postpartum**						
	Ao_Vmáx_(Doppler)	101.0	14.2	103.0	13.7	0.476
	ET_(msec)	195.0	22.3	192.0	15.8	0.572
	HR	140.0	15.9	142.0	12.1	0.393
	IVCT_(msec)	44.8	11.0	45.5	12.1	0.764
	IVRT_(msec)	48.5	8.38	51.5	10.3	0.159
	LVOT_d	7.95	1.13	8.16	1.05	0.362
	MPI	44.8	11.1	45.8	12.1	0.693
	MV_A	74.1	15.9	81.4	19.8	0.0756
	MV_E	92.8	17.6	89.0	14.4	0.278
	TAPSE_(mm)	12.0	1.61	11.7	1.51	0.392
	TV_A	65.1	18.4	70.0	16.5	0.211
	TV_E	67.7	13.8	66.2	13.1	0.621

Results are presented as the mean (standard deviation) for continuous variables. Comparisons between groups were carried out using the *t*-test for continuous variables and the chi-square test or Fisher’s exact test for qualitative variables. Ao Vmáx (Doppler): maximal aortic blood flow velocity; ET: ejection time; HR: heart rate; IVCT: isovolumetric contraction time; IVRT: isovolumetric relaxation time; LVOT d: left ventricular outflow tract diameter; MPI: myocardial performance index; MV A: mitral valve A-wave velocity; MV E: mitral valve E-wave velocity; TAPSE: tricuspid annular plane systolic excursion; TV A: tricuspid valve A-wave velocity; TV E: tricuspid valve E-wave velocity.

**Table 3:** Linear mixed-effects model for aortic peak velocity (Ao_Vmáx) measured by Doppler according to treatment and time.

Fixed Effect	Estimate	Std. Error	df	t Value	*p* Value
(Intercept)	77.265	1.916	264.605	40.324	<0.001
Treatment 1	−5.634	2.922	264.605	−1.928	0.0549
time 1	6.070	2.514	185.033	2.415	0.0167
time 2	23.677	2.625	192.140	9.022	<0.001
Treatment 1:time 1	7.013	3.787	182.580	1.852	0.0657
Treatment 1:time 2	7.971	3.939	189.228	2.023	0.0445

Treatment 1 corresponds to the ursodeoxycholic acid group.

**Table 4:** Linear mixed-effects model for mitral A-wave velocity (MV_A) measured by Doppler according to treatment and time.

Fixed Effect	Estimate	Std. Error	df	t Value	*p* Value
(Intercept)	50.546	1.635	274.874	30.911	<0.001
Treatment 1	−2.258	2.510	274.881	−0.899	0.3693
time 1	−0.205	2.337	187.893	−0.088	0.9300
time 2	23.504	2.476	200.544	9.494	<0.001
Treatment 1:time 1	1.338	3.538	186.178	0.378	0.7056
Treatment 1:time 2	9.583	3.709	197.015	2.583	0.0105

Treatment 1 corresponds to the ursodeoxycholic acid group.

## Data Availability

Catalina De Paco Matallana is a guarantor of this study, and, as such, had full access to all of the data in the study and takes responsibility for the integrity of the data and the accuracy of the data analysis.

## Supplementary Materials

The following supporting information can be downloaded at: https://www.mdpi.com/article/10.3390/jcm14207366/s1, Table S1: Linear mixed-effects model for ejection time (ET, msec) measured by Doppler according to treatment and time; Table S2: Linear mixed-effects model for fetal heart rate (HR) according to treatment and time; Table S3: Linear mixed-effects model for isovolumetric contraction time (IVCT, msec) measured by Doppler according to treatment and time; Table S4: Linear mixed-effects model for isovolumetric relaxation time (IVRT, msec) measured by Doppler according to treatment and time; Table S5: Linear mixed-effects model for left ventricular outflow tract diameter (LVOT_d) measured by Doppler according to treatment and time; Table S6: Linear mixed-effects model for myocardial performance index (MPI) measured by Doppler according to treatment and time; Table S7: Linear mixed-effects model for tricuspid annular plane systolic excursion (TAPSE, mm) measured by Doppler according to treatment and time.
