# Supplementary material for: The Impact of Ursodeoxycholic Acid on Fetal Cardiac Function in Women with Gestational Diabetes Mellitus: A Randomized Controlled Study (GUARDS Trial)
Source: J Clin Med. 2025 Oct 17;14(20):7366. doi: 10.3390/jcm14207366 (PMC12565255; doi:10.3390/jcm14207366)
Supplement: Supplementary file 1 [file jcm-14-07366-s001.zip › jcm-3881491-supplementary.pdf]

Table S1. Linear mixed-effects model for ejection time (ET, msec) measured by Doppler according to treatment and time

Fixed effect	Estimate	Std. Error	df	t value	p value
(Intercept)	164.965	2.463	280.000	66.980	<0.001
Treatment1	0.291	3.756	280.000	0.077	0.9380
time1	2.790	3.548	280.000	0.786	0.4320
time2	29.796	3.685	280.000	8.085	<0.001
Treatment1:time1	-1.883	5.354	280.000	-0.352	0.7250
Treatment1:time2	-2.657	5.543	280.000	-0.479	0.6320

Treatment 1 corresponds to the ursodeoxycholic acid group

Table S2. Linear mixed-effects model for fetal heart rate (HR) according to treatment and time.

Fixed effect	Estimate	Std. Error	df	t value	p value
(Intercept)	143.860	1.711	269.417	84.076	<0.001
Treatment1	-1.929	2.609	269.417	-0.739	0.4603
time1	-4.272	2.284	186.677	-1.871	0.0630
time2	-4.063	2.383	194.078	-1.705	0.0898
Treatment1:time1	3.063	3.442	184.200	0.890	0.3747
Treatment1:time2	4.726	3.577	191.114	1.321	0.1881

Treatment 1 corresponds to the ursodeoxycholic acid group

Table S3. Linear mixed-effects model for isovolumetric contraction time (IVCT, msec) measured by Doppler according to treatment and time.

Fixed effect	Estimate	Std. Error	df	t value	p value
(Intercept)	40.439	1.283	272.917	31.511	<0.001
Treatment1	3.445	1.957	272.917	1.760	0.0795
time1	1.007	1.740	186.329	0.579	0.5633
time2	4.243	1.814	194.087	2.339	0.0203

Treatment1:time1	-3.449	2.623	183.811	-1.315	0.1901
Treatment1:time2	-2.696	2.724	191.046	-0.990	0.3235

Treatment 1 corresponds to the ursodeoxycholic acid group

Table S4. Linear mixed-effects model for isovolumetric relaxation time (IVRT, msec) measured by Doppler according to treatment and time.

Fixed effect	Estimate	Std. Error	df	t value	p value
(Intercept)	51.474	1.396	279.323	36.878	<0.001
Treatment1	3.829	2.128	279.323	1.799	0.0731
time1	6.465	1.974	190.403	3.274	0.0013
time2	-2.955	2.053	198.947	-1.439	0.1517
Treatment1:time1	-2.163	2.979	187.857	-0.726	0.4687
Treatment1:time2	-0.836	3.087	195.798	-0.271	0.7869

Treatment 1 corresponds to the ursodeoxycholic acid group

Table S5. Linear mixed-effects model for left ventricular outflow tract diameter (LVOT_d) measured by Doppler according to treatment and time.

Fixed effect	Estimate	Std. Error	df	t value	p value
(Intercept)	4.649	0.111	275.905	41.722	<0.001
Treatment1	-0.031	0.170	275.905	-0.180	0.8580
time1	1.483	0.154	186.371	9.606	<0.001
time2	3.291	0.161	194.554	20.480	<0.001
Treatment1:time1	-0.059	0.233	183.826	-0.255	0.7990
Treatment1:time2	0.259	0.243	192.290	1.066	0.2880

Treatment 1 corresponds to the ursodeoxycholic acid group

Table S6. Linear mixed-effects model for myocardial performance index (MPI) measured by Doppler according to treatment and time.

Fixed effect	Estimate	Std. Error	df	t value	p value
(Intercept)	40.616	1.261	266.207	32.209	<0.001
Treatment1	3.131	1.923	266.207	1.628	0.1047
time1	0.640	1.663	186.273	0.385	0.7007
time2	4.090	1.735	193.431	2.357	0.0194
Treatment1:time1	-3.084	2.505	183.822	-1.231	0.2198
Treatment1:time2	-2.140	2.605	190.518	-0.821	0.4124

Treatment 1 corresponds to the ursodeoxycholic acid group

Table S7. Linear mixed-effects model for tricuspid annular plane systolic excursion (TAPSE, mm) measured by Doppler according to treatment and time.

Fixed effect	Estimate	Std. Error	df	t value	p value
(Intercept)	5.861	0.339	280.000	17.278	<0.001
Treatment1	-0.080	0.517	280.000	-0.154	0.8774
time1	1.410	0.489	280.000	2.886	0.0042
time2	6.149	0.508	280.000	12.114	<0.001
Treatment1:time1	1.306	0.738	280.000	1.771	0.0777
Treatment1:time2	-0.214	0.763	280.000	-0.280	0.7799

Treatment 1 corresponds to the ursodeoxycholic acid group
